# Parametric Optimization and Performance Analysis of an Internally Cooled Structured Reactor for CO_2_ Direct Air Capture via Temperature–Vacuum Swing Adsorption

**DOI:** 10.3390/molecules31111976

**Published:** 2026-06-05

**Authors:** Jiale Zheng, Wenqi Fan, Chuanruo Yang, Ming Xue, Zhexuan An, Xinglei Zhao, Xingchun Li, Aiguo Zhou, Liang Huang

**Affiliations:** 1 State Key Laboratory of Petroleum Pollution Control, Beijing 102206, China; zhengjiale@cnpc.com.cn (J.Z.); fanwenqi97@163.com (W.F.); yangchuanruo@cnpc.com.cn (C.Y.); mxue@cnpc.com.cn (M.X.); anzhexuan@cnpc.com.cn (Z.A.); zhaoxinglei@cnpc.com.cn (X.Z.); 2 China National Petroleum Corporation Research Institute of Safety and Environmental Technology, Beijing 102206, China; 3 College of Environmental Science and Engineering, Beijing Forestry University, Beijing 100083, China; l_huang@bjfu.edu.cn

**Keywords:** direct air capture, temperature–vacuum swing adsorption, simulation

## Abstract

Direct air capture (DAC) based on adsorption is a promising negative-emission technology owing to its operational flexibility, modular deployment potential, and comparatively low regeneration temperature. In this study, a dynamic three-dimensional mathematical model was developed to investigate a structured adsorption-based DAC reactor operating under a temperature–vacuum swing adsorption cycle. The model couples heat and mass transfer among the gas, adsorbent, metal structure, and heat-transfer fluid and was used to evaluate the temporal and spatial evolution of temperature and CO_2_ adsorption capacity during adsorption and regeneration. The effects of internal cooling, heat-source temperature, and vacuum pressure on cyclic performance were systematically analyzed. The results show that introducing an internal cooling source significantly accelerates adsorbent-bed cooling and increases the cyclic working capacity by approximately 10%. Parametric simulations indicate that higher regeneration temperature and lower vacuum pressure enhance CO_2_ desorption, with optimal performance achieved at a heat-source temperature of 90 °C and a vacuum pressure of 1 kPa. Under these conditions, the DAC system reaches an annual CO_2_ productivity of 125 tCO_2_·year^−1^, with mechanical and thermal energy consumptions of 4.72 and 11.91 GJ·tCO_2_^−1^, respectively. This work provides a useful modeling framework for reactor design and operating-parameter optimization in adsorption-based DAC systems.

## 1. Introduction

Based on the annual report of the Global Monitoring Laboratory (GML) of the National Oceanic and Atmospheric Administration (NOAA), the global average atmospheric CO_2_ concentration reached a record high of 419.3 ppm in 2023, with an annual increase of 2.8 ppm. This marked the 12th consecutive year that the atmospheric CO_2_ level has increased by more than 2 ppm [1]. The development of CO_2_-negative technologies is urgently needed [2].

Direct Air Capture (DAC) is a technology that captures CO_2_ directly from ambient air using specific materials [3]. There are two main categories: absorption [4,5] and adsorption [6,7]. The absorption method typically requires high-grade thermal energy for regeneration [8,9], whereas adsorption-based DAC operates at significantly lower regeneration temperatures [10,11]. As a result, adsorption-based DAC is expected to achieve lower operating costs in the range of 29~91 $/tCO_2_ [12,13]. The CO_2_ captured by DAC can be subsequently converted, stored, or utilized, enabling DAC to mitigate climate change impacts by addressing non-point emission sources without reliance on emission-site control [14,15,16]. Moreover, DAC can be deployed globally and is inherently sustainable, as atmospheric CO_2_ represents an abundant and widely available carbon resource, making it a potential cornerstone of a future carbon-cycle economy.

Compared with other CO_2_ abatement technologies, DAC systems require relatively limited land and water resources and are not geographically constrained [17]. Nevertheless, the extremely low CO_2_ partial pressure in the atmosphere (~420 ppm) [1] poses significant challenges to adsorption performance and operating costs. Therefore, further developments and breakthroughs in DAC are required. Current research primarily focuses on three directions: material improvement, cycle design, and system optimization. Significant efforts have been devoted to the development of high-performance adsorbents, including zeolites [18,19], metal-organic frameworks (MOFs) [20,21], silica [22], activated carbon [23], silica gel [24,25], etc. In parallel, various DAC process cycles have been proposed, such as temperature swing adsorption (TSA) [23,26,27], temperature vacuum swing adsorption (TVSA) [24,28], steam-assisted temperature swing adsorption (S-TSA) [29,30], steam-assisted temperature vacuum swing adsorption (S-TVSA) [31,32,33], moisture swing adsorption (MSA) [34,35]. In addition, different reactor configurations (fixed bed [36,37], fluidized bed [26], moving bed [27,38], rotary bed [23,39], etc.) have been developed to accommodate diverse adsorbents and cycling processes.

Prototyping and subsequent scale-up of DAC systems require substantial investments in manpower, capital, and operating costs (typically 60–230 $/tCO_2_ [4,27,29,34]). Simulation is an effective tool for assessing the efficiency, energy consumption, economic performance, and environmental impacts of DAC systems. Simulation studies commonly involve hydrodynamic simulation, thermodynamic simulation, and system optimization. In this work, a dynamic three-dimensional mathematical method is developed to simulate the adsorption-desorption behavior of an adsorption-based DAC system operating under a TVSA cycle. Given that current research has relatively few studies on DAC reactors, this paper proposes a reactor with an internal heat exchange mechanism, aiming to fill the research gap. The model is constructed to evaluate the system productivity and energy consumption, providing insight into the performance and optimization of DAC processes.

## 2. Results and Discussions

### 2.1. No Internal Cooling Sources

**Figure 1:**
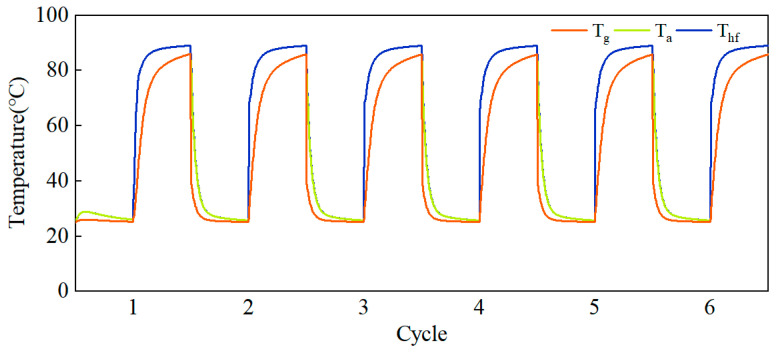
Method for solving the model.

A simulation of the current reactor structure is performed under an ambient temperature of 25 °C and a heat source temperature of 90 °C. The temperature distribution of heat exchange fluid, adsorbent, and gas in the reactor with time is presented in Figure 1. In the initial adsorption stage, the adsorbent is rapidly blown by the incoming air to cool down, and the temperature of the adsorbent drops sharply with the gas temperature. During the desorption phase, the heat exchange fluid heats the adsorbent, and the temperature of the adsorbent increases with the temperature of the heat exchange fluid. Maintain the heat exchange temperature difference within 3 °C at the end of desorption. The overall response speed of temperature is fast, and the efficient temperature state switch is realized.

**Figure 2:**
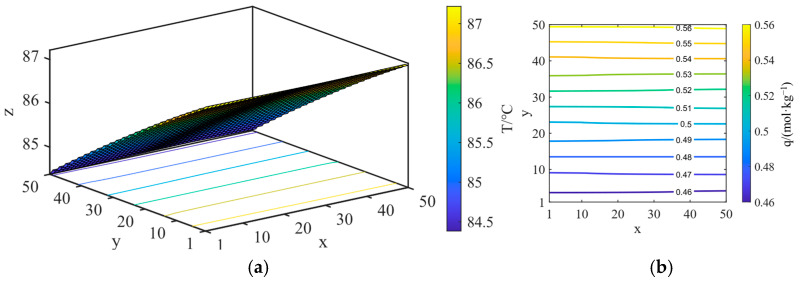
Spatial distribution of temperature and adsorption capacity at desorption endpoint. (**a**) Temperature at desorption endpoint; (**b**) Adsorption capacity at desorption endpoint.

The spatial distribution of desorbed end-point adsorbent bed temperature and adsorption capacity is depicted in Figure 2. The desorption terminal temperature distribution in a single adsorbent sheet is uniform and close to the heat source temperature. The maximum and minimum temperatures in space differ by less than 3 °C. The spatial state of desorption end-point adsorption in a single adsorbent sheet is similar. After desorption, the valley value of adsorption was about 0.51 mol·kg^−1^, and the maximum difference of the valley value of adsorption in space was about 0.1 mol·kg^−1^.

### 2.2. With Internal Cooling Source

**Figure 3:**
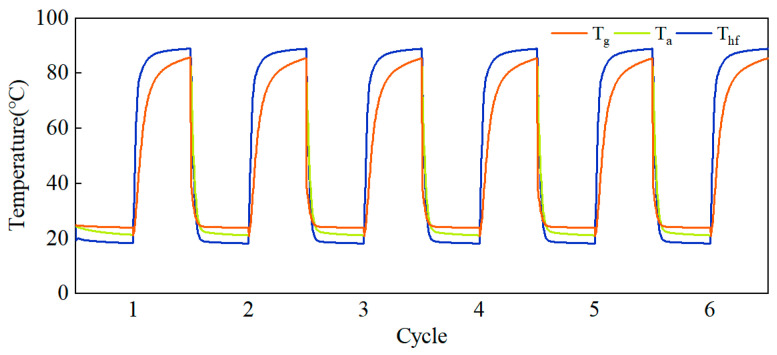
Time distribution of temperature in the reactor with internal cooling source.

Further, cooling water is supplied to the heat exchange structure as an internal cooling source during the adsorption stage. Under an ambient temperature of 25 °C, a cold source temperature of 15 °C, and a heat source temperature of 90 °C, the temperature-time distribution of heat exchange fluid, adsorbent, and gas in the reactor is shown in Figure 3. At the beginning of the adsorption phase, the desorbed cooling water will rapidly cool the adsorbent at a temperature close to 90 °C to make its temperature close to the cold source temperature. The difference between the two temperatures is about 3 °C. Compared with the reactor without an internal cooling source, the temperature of the adsorbent bed in the reactor decreases significantly after increasing internal cooling.

**Figure 4:**
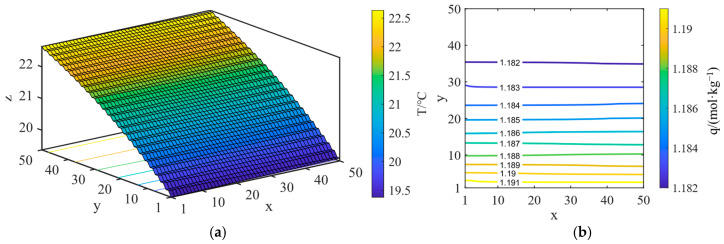
Spatial distribution of temperature and adsorption capacity at adsorption endpoint with internal cooling source. (**a**) Temperature at adsorption endpoint; (**b**) Adsorption capacity at adsorption endpoint.

The spatial distribution of adsorption terminal temperature and adsorption capacity after increasing the internal cooling source is illustrated in Figure 4. It can be seen that the temperature in the reactor drops rapidly when entering the adsorption stage, and the temperature response is significantly accelerated. The temperature in a single adsorbent sheet is still evenly distributed. The addition of an internal cooling source brings new changes to the spatial distribution of temperature in the adsorption process, and the isotherm is arranged in parallel with the heat exchange tube. At the end of adsorption, the average temperature of the adsorbent flake in the reactor is 21 °C. Compared to the case without an internal cooling source, the temperature of the adsorbent flake inside the reactor drops markedly.

As depicted in Figure 4b, the amount of adsorbent CO_2_ adsorbed by a single adsorbent sheet in the reactor corresponds to the temperature distribution. The adsorption capacity contour is parallel to the flow direction of the heat exchange fluid in the reactor. The adsorption shows a monotonic decreasing trend along the fluid flow direction. The difference between the highest point and the lowest point of the adsorption capacity at the end point was controlled within 0.01 mol·kg^−1^ and higher than 1.18 mol·kg^−1^.

**Figure 5:**
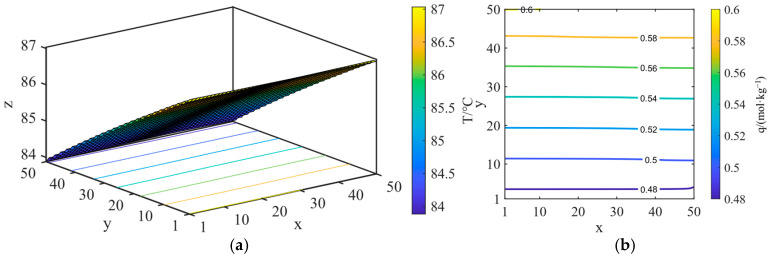
Spatial distribution of temperature and adsorption capacity at desorption endpoint with internal cooling source. (**a**) Temperature at desorption endpoint; (**b**) Adsorption capacity at desorption endpoint.

The spatial distribution of desorption terminal temperature and adsorption capacity is summarized in Figure 5. The spatial distribution of the desorption terminal temperature of a single adsorbent sheet is similar to that before the addition of the internal cooling source, with a temperature difference of about 3 °C. Uniform spatial distribution of adsorption capacity. The spatial adsorption difference was about 0.12 mol·kg^−1^ and was not higher than 0.6 mol·kg^−1^. The results indicate that the utilization of adsorbents in different areas is the same, and the adsorption capacity is fully utilized. The cycle operating capacity is 0.647 mol·kg^−1^, which is increased by approximately 10% compared with that before increasing the internal cooling source.

### 2.3. Heat Source Temperature

The simulation calculation shows that the response speed of the adsorbent bed temperature is very fast under the current reactor structure. Evacuation and preheating time shall be set as 5 min, and the time of the whole adsorption process and regeneration process shall be controlled within 50 min, and the follow-up performance parameters are explored with a cycle of 100 min.

**Figure 6:**
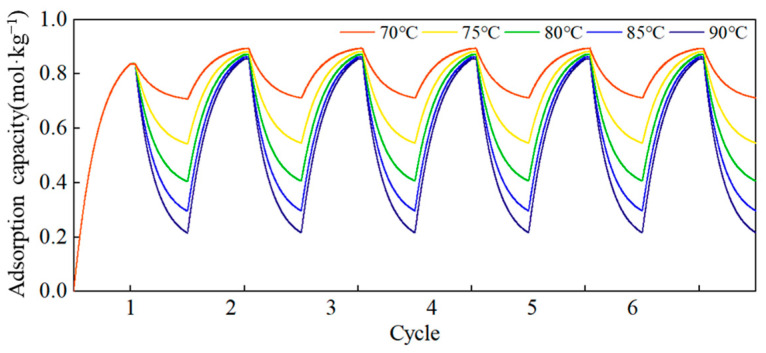
Change of circulating adsorption capacity with heat source temperature.

Probe into the influence of heat source temperature on cycle performance. Control the vacuum pressure at the time of gas production by air extraction at 1 kPa, and inject heat exchange fluid with different heat source temperatures. The calculation results are displayed in Figure 6. As the heat source temperature decreases, the desorption of CO_2_ gradually slows down, and the amount of CO_2_ desorbed in the same cycle decreases as well.

**Figure 7:**
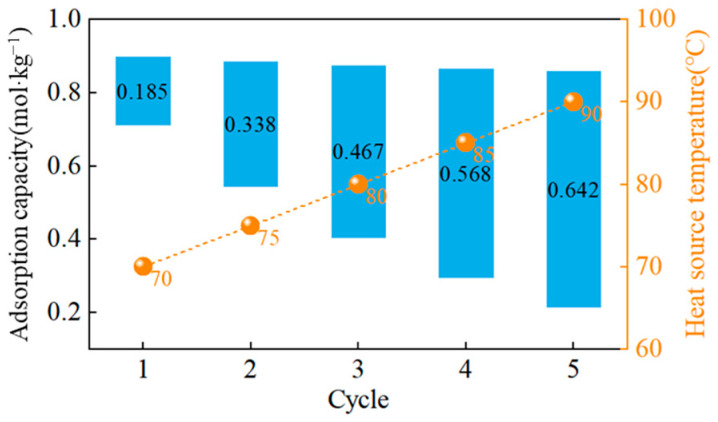
Change of circulating working capacity with heat source temperature.

The variation of cycle operating capacity with heat source temperature is shown in Figure 7. Desorption is most thorough when the heat source temperature is 90 °C. The peak value and valley value of cyclic adsorption are 0.857 mol·kg^−1^ and 0.215 mol·kg^−1^, respectively, with a difference of 0.642 mol·kg^−1^ representing the cyclic working capacity. The gradual increase in the adsorption valley value leads to a gradual increase in the adsorption peak value. However, the difference between the two is gradually reduced, that is, the circulation capacity decreases significantly as the heat source temperature drops. At the heat source temperature of 70 °C, the cycle operating capacity is only 0.185 mol·kg^−1^.

### 2.4. Vacuum Pressure

**Figure 8:**
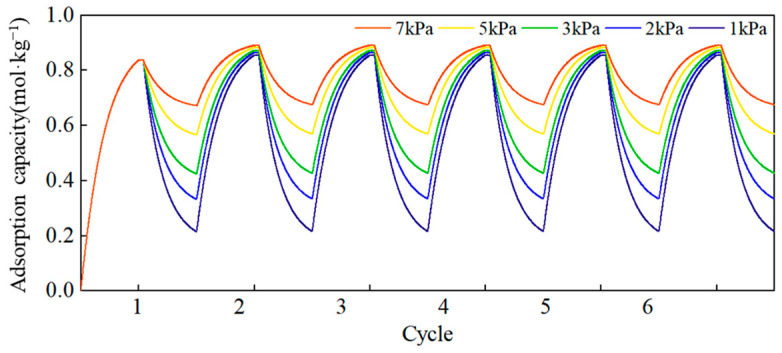
Change of circulating adsorption capacity with vacuum pressure.

Probe into the Influence of Vacuum Pressure on Cyclic Performance. Maintain the heat source temperature of 90 °C, and control the vacuum pressure at the extraction and gas production stage to be 1, 2, 3, 5, and 7 kPa, respectively. The calculation results are indicated in Figure 8. The desorption of CO_2_ decreases with increasing vacuum pressure.

**Figure 9:**
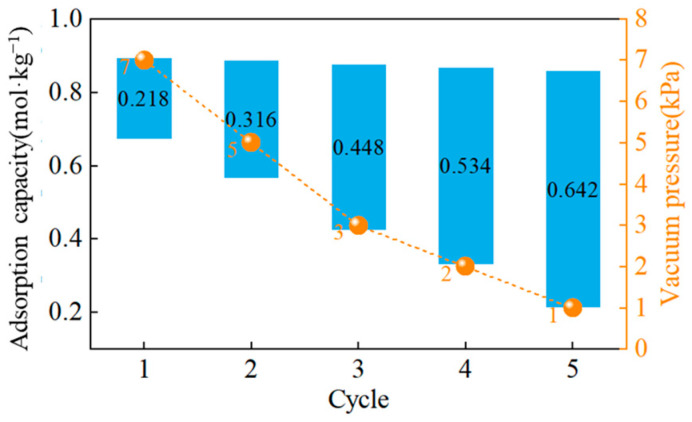
Change of circulating working capacity with vacuum pressure.

The variation of cycle operating capacity with vacuum pressure is summarized in Figure 9. The most thorough desorption occurs at a vacuum pressure of 1 kPa, with a circulating operating capacity of 0.642 mol·kg^−1^. With the increase in vacuum pressure, the “adsorption-desorption” equilibrium gradually shifts towards the adsorption direction, leading to circulation. The working capacity decreases significantly with the increase in vacuum pressure. At the vacuum pressure of 7 kPa, the cycle operating capacity is only 0.218 mol·kg^−1^, which is reduced by 66% compared to the 1 kPa vacuum pressure.

Based on the above analysis, under the premise of the same adsorbent mass and air flow, with a heat source of 90 °C and a vacuum pressure of 1 kPa, the TVSA cycle mode has the highest cyclic output. At this time, the cyclic adsorption performance of the DAC system is the best.

### 2.5. Productivity and Energy Consumption

Productivity is the amount of CO_2_ that the system can produce per unit of time. It reflects the production efficiency of DAC technology and is an important index to evaluate its economic benefits and application value. When the unit of production rate is tCO_2_·year^−1^, it represents the mass of CO_2_ captured and produced by the system every year, which is calculated by Equation (1):

(1) $$Productivity=\frac{\int_{0}^{t_{\mathrm{day}}}M_{CO_{2}}C_{\mathrm{pro}}V_{f,\mathrm{pro}}dt}{m_{a}t_{\mathrm{day}}}$$

where *C*_pro_ is the amount concentration of CO_2_ in the product gas, mol·m^−3^. *m*_a_ is the mass of adsorbent, kg. Through calculation, the productivity under the current scale is 125 tCO_2_·year^−1^.

Unit energy consumption refers to the energy consumption of the DAC system per unit of CO_2_, and the unit is GJ·tCO_2_^−1^. It directly reflects the energy utilization efficiency and system operation cost of DAC technology. Based on the specific development mode of the DAC cycle, the unit energy consumption is divided into the energy consumption of the adsorption process and the energy consumption of the regeneration process. Among them, the energy consumption of the adsorption process is mainly mechanical energy to overcome the flow resistance. The energy consumption of the regeneration process includes the vacuum mechanical energy of the evacuation process and the gas production process, as well as the heat energy of the regeneration process.

#### 2.5.1. Energy Consumption of Adsorption Process

The mechanical energy consumption during adsorption refers to the energy consumption required by the fan to push the air through the adsorbent bed of the reactor. Ignoring the energy conversion loss of the fan, the product of air volume flow and pressure drop of the adsorption bed can quantify the flowing mechanical energy. The unit energy consumption *E*_b_ (GJ·tCO_2_^−1^) is calculated by Equation (2):

(2) $$E_{b}=5.09\times 10^{-7}\frac{\Delta P}{x_{CO_{2}}CR}$$

where Δ*P* is the pressure drop across the bed, Pa. *CR* is the CO_2_ capture rate for the adsorption process, %. *x*_CO_2__ is the mole fraction of CO_2_ in the ambient air, %. The flow mechanical energy of the adsorption process is closely related to the bed pressure drop, and the pressure drop is generally calculated by Equation (3) proposed by Ergun:

(3) $$\Delta P=150\frac{v\mu (1-\varepsilon {)}^{2}h}{{\left(\phi d_{p}\right)}^{2}\varepsilon^{3}}+1.75\frac{v^{2}\rho (1-\varepsilon )h}{\phi d_{p}\varepsilon^{3}}$$

where *v*, *ρ* and *μ* are the apparent velocity (m·s^−1^), density (kg·m^−3^) and dynamic viscosity (Pa·s) of the flow, respectively. *D*_p_ and *ϕ*, respectively, represent the particle size (m) and the sphericity (*ϕ* = 1) of the adsorbent. And *h* is the bed void ratio and bed thickness (m), respectively.

Under the current ultra-thin bed thickness, air is purged at a flow rate of approximately 0.2 m·s^−1^ during the adsorption process. The pressure drop across the bed is around 222 kPa. The energy consumption required to overcome the flow resistance during adsorption is 0.90 GJ·tCO_2_^−1^.

#### 2.5.2. Vacuum Mechanical Energy for Evacuation and Desorption Process

At the end of the adsorption phase, the vacuum pump is normally started to extract the residual air from the reactor. In order to discharge the residual gas in the system, the energy consumed by the vacuum pump is called the vacuum mechanical energy in the evacuation process. During desorption, the vacuum pump will be started to create a vacuum environment for the reactor to reduce the partial pressure of CO_2_ around the adsorbent, promote desorption, and collect product gas. The energy consumption of the pump in this process is called the vacuum mechanical energy in the gas production process. In actual operation, the efficiency of the vacuum pump shall be considered. By Equation (4):

(4) $$E_{v}=-\int_{V_{\mathrm{ini}}}^{V_{\mathrm{fin}}}PdV/m_{CO_{2}}$$

where *V*_ini_ and *V*_fin_ are the initial and final volumes, respectively, m^3^. *m*_CO_2__ is the mass of CO_2_, kg.

The vacuum mechanical energy corresponding to the emptying process and desorption process are 0.11 and 3.70 GJ·tCO_2_^−1^, respectively.

#### 2.5.3. Thermal Energy from Regeneration Process

In adsorption DAC, the heat energy of the regeneration process is one of the core energy consumptions. In the TVSA cycle, a certain amount of heat is supplied to the adsorbents at the final state of adsorption to realize the desorption of adsorbents. In actual operation, additional heat shall be provided to meet the sensible heat of the adsorbent, reactor, etc., and to minimize unnecessary heat loss. Other heat losses are negligible, and the required heat energy of the regeneration process per unit CO_2_ (*E*_re_, GJ·t^−1^) is calculated from Equation (5):

(5) $$E_{\mathrm{re}}=Q_{\mathrm{lh}}+Q_{\mathrm{sh}}$$

where *Q*_lh_ and *Q*_sh_ are the heat of desorption and the sensible heat, respectively, GJ·t^−1^. The heat of desorption is directly related to the enthalpy change of reaction. Generally, CO_2_ adsorbents have a certain adsorption effect on H_2_O, which leads to unavoidably synchronous adsorption/desorption cycle for H_2_O when DAC operates the TVSA cycle.

(6) $$Q_{\mathrm{lh}}=\frac{m_{CO_{2}}\Delta H_{CO_{2}}+m_{H_{2}O}\Delta H_{H_{2}O}}{m_{CO_{2}}}$$

where *m*_*CO*_2__ and Δ*H*_*CO*_2__ represent the mass (t) and the enthalpy change of reaction (GJ·t^−1^) for the desorbed CO_2_, respectively; *m*_*H*_2_*O*_ and Δ*H*_*H*_2_*O*_ represent the mass (t) and the enthalpy change (GJ·t^−1^) for the desorbed H_2_O, respectively.

**Figure 10:**
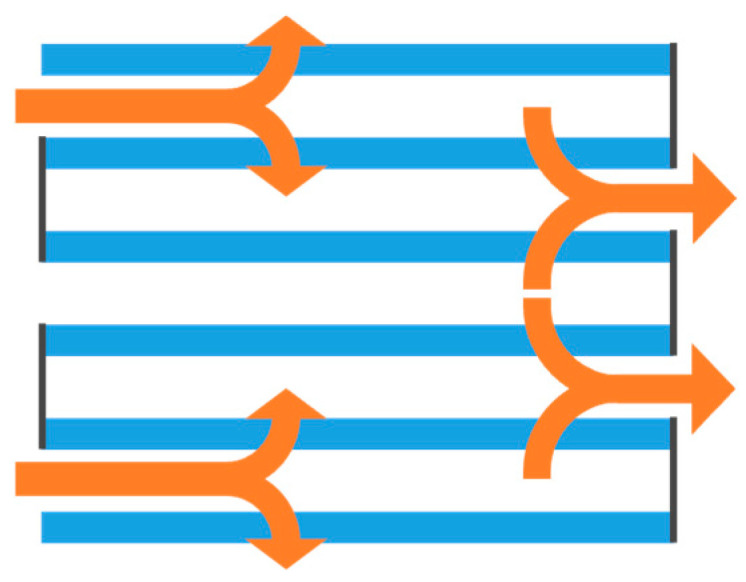
Air path of DAC reactor.

The calculation of sensible heat is represented by Equation (7), which is closely related to the specific heat capacities of the adsorbent and reactor.

(7) $$Q_{\mathrm{sh}}=\frac{\sum_{i}\int_{T_{\mathrm{ini}}}^{T_{\mathrm{fin}}}m_{i}c_{p,i}dT}{m_{CO_{2}}}$$

where *m*_i_ and *c*_p,i_ represent the mass and specific heat capacity of the adsorbent, reactor (as shown in Figure 10) and gas, respectively. *T*_ini_ and *T*_fin_ are the initial and final temperatures, respectively, K.

The heat consumption for the desorption process is 11.91 GJ·tCO_2_^−1^, in which water is adsorbed or desorbed by the adsorbent at a molar amount 3.8 times that of CO_2_. In this work, the total heat of adsorption is about 6.18 GJ·tCO_2_^−1^, and the specific proportion of the adsorption heat of water is about 60%. Besides, the sensible heat of the reactor is 1.6 GJ·tCO_2_^−1^, which includes the whole metal structure. The remaining heat consumption is the sensible heat of the adsorbent.

## 3. Physical Model of DAC Reactor

The internal structure of the reactor is shown in Figure 10. Keep the adsorbent sheets parallel, and arrange wind shields at intervals on both the upstream and downstream sides of the air channels. Ambient air enters the reactor from one side, passes through a single layer of adsorbent, and exits from the other side. The problem of too high mechanical energy in the adsorption process caused by adsorbent accumulation is effectively solved [40,41,42], and the volume of the reactor is tightly controlled.

**Figure 11:**
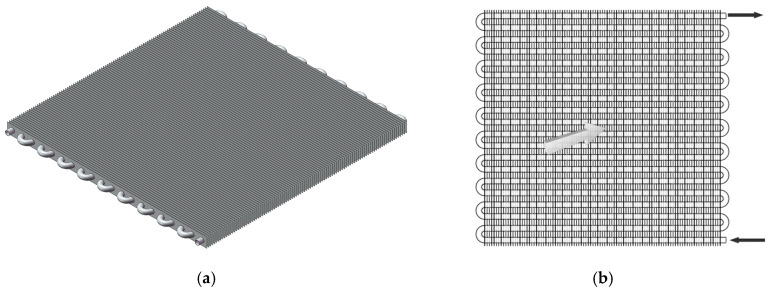
Internal structure of a single adsorbent sheet layer of DAC reactor. (**a**) The graphic model; (**b**) The top view.

The internal structure of an individual adsorbent sheet is presented in Figure 11. An S-type copper tube is vertically welded with parallel aluminum fins, with solid adsorbent distributed on the external surfaces. A heat-transfer fluid circulates through the tube to supply or remove thermal energy as required. Meanwhile, the airflow vertically sweeps the outside for heat and mass exchange. The reactors operate in an alternating cycle of adsorption and desorption.

Record the structure parameters in Table 1.

## 4. Mathematical Model

The reactor is designed as a square structure with parallel heat exchanger tubes and welded metal fins. A heat-transfer fluid flows through the tubes, while the adsorbent is distributed within the chamber outside the tubes. The temperature fields of the four substances—air, adsorbent, metal, heat-transfer fluid—are coupled to each other. The adsorbate migrates between the air and the adsorbent.

To simplify the mathematical model, the following assumptions are adopted:

(1) The system is well insulated, and heat exchange with the external environment is neglected.
(2) Uniform distribution of adsorbent and uniform pressure inside the reactor chamber.
(3) Constant physical properties, and the fluid is considered as one-dimensional inviscid flow.
(4) Neglect the heat transfer of the metal and the adsorbent along with the fluid flow direction.
(5) Internal heat transfer within the adsorbent is neglected, and no degradation of adsorbent performance occurs over time.
(6) During desorption, the reactor cavity contains an ideal gas with a uniform mixture of components.
(7) Neglect the switching time of the system; maintain the limit vacuum state stably.

### 4.1. Governing Equations

A temperature vacuum-swing adsorption cycle (TVSA) is applied for the DAC system, consisting of four steps: adsorption-evacuation-heating-desorption [43]. In the adsorption step, a four-layer heat and mass transfer model of air-adsorbent-metal-heat transfer fluid is constructed. In the desorption step, the gas is very dilute due to the extreme vacuum in the reactor chamber. A three-layer heat and mass transfer model of adsorbent-metal-heat transfer fluid was used instead. The temperature of the gas is considered to be the same as that of the adsorbent.

Combining the first law of thermodynamics and the above assumptions, a set of energy control equations for the TVSA cycle is given. Firstly, the energy balance of the gas in the reactor is expressed as Equation (8):

(8) $$M_{gas\;}c_{p,gas\;}\frac{\partial T_{gas\;}}{\partial t}+m_{f,gas\;}l_{gas\;}c_{p,gas\;}\frac{\partial T_{gas}}{\partial z}=(KF{)}_{gas,ads\;}\left(T_{ads\;}-T_{gas\;}\right)$$

The first term on the left is the change of energy of the gas. The second term on the left is the energy carried by the gas flow. The right side is the energy flow achieved by heat transfer, i.e., heat exchange between gas and adsorbent.

The energy balance of the adsorbent is expressed by Equation (9):

(9) $$\begin{array}{cc} (m_{ads}c_{p,ads}+m_{ads}q_{CO_{2}}c_{p,CO_{2}})\frac{\partial T_{ads}}{\partial t} & =m_{ads}\frac{dq_{CO_{2}}}{dt}\Delta H_{CO_{2}}+m_{ads}\frac{dq_{CO_{2}}}{dt}c_{p,CO_{2}}(T_{gas}-T_{ads})\\ & +{\left(KF\right)}_{gas,ads}(T_{gas}-T_{ads})+{\left(KF\right)}_{ads,met}(T_{met}-T_{ads}) \end{array}$$

where the left-hand term represents the energy change of the adsorbent. The first term on the right gives the heat of adsorption. The last two terms represent the heat exchange with gas and metal, respectively.

The energy control equation for the metal is shown in Equation (10):

(10) $$m_{met}c_{p,met}\frac{\partial T_{met}}{\partial t}=(KF{)}_{ads,met}(T_{ads}-T_{met})+(KF{)}_{met,hf}(T_{hf}-T_{met})$$

Similarly, the left-hand and right-hand terms represent the energy change and heat exchange of the metal, respectively.

Finally, the following Equation (11) gives the energy balance of the heat exchange fluid.

(11) $$m_{hf}c_{p,hf}\frac{\partial T_{hf}}{\partial t}+m_{f,hf}l_{hf}c_{p,hf}\frac{\partial T_{hf}}{\partial y}=(KF{)}_{met,hf}(T_{met}-T_{hf})$$

where the left two terms are the energy change in the heat transfer fluid and the net energy flow out of the control volume, and the right side is the net heat flow achieved by heat exchange.

The mass balance is defined by Equation (12).

(12) $$m_{gas}\frac{\partial \omega_{CO_{2}}}{\partial t}=m_{f,gas}l_{gas}\frac{\partial \omega_{CO_{2}}}{\partial z}-m_{gas}\frac{dq_{m,CO_{2}}}{dt}$$

### 4.2. Auxiliary Equations

Add other equations to achieve a closed system to solve. Amine adsorbents not only exhibit better adsorption capacity but also lower regeneration temperature. The performance of the amine adsorbent used in this paper has been summarized in the CO_2_ adsorption isotherm equation and the adsorption kinetic equation.

The adsorption isotherm equation of CO_2_ is represented by the following equations [44]. The values of the parameters involved are given in Table 2.

(13) $$q_{\mathrm{eq}}=q_{p}+q_{c}\eta =\frac{q_{\infty ,p}(T)b_{p}(T)P}{{\left[1+(b_{p}(T)P{)}^{\tau (T)}\right]}^{1/\tau (T)}}+\frac{q_{\infty ,c}b_{\mathrm{ref},c}P}{1+b_{\mathrm{ref},c}P}\exp\left(\frac{-E_{a}}{RT}\right)$$

(14) $$q_{\infty ,p}(T)=q_{\infty ,\mathrm{ref}}\exp[\chi (1-\frac{T}{T_{\mathrm{ref}}})]$$

(15) $$b_{p}(T)=b_{\mathrm{ref},p}\exp(\frac{-\Delta H}{RT})$$

(16) $$\tau (T)=\tau_{\mathrm{ref}}+\alpha (1-\frac{T_{\mathrm{ref}}}{T})$$

where *q*_∞,p_(*T*) is the maximum adsorption capacity of CO_2_. *Q*_∞,ref_ is the maximum adsorption capacity of CO_2_ at reference temperature *T*_ref_. *b*_p_(*T*) is the CO_2_ adsorption coefficient defined by the constant *b*_ref_ and the heat of adsorption Δ*H*. *R* is the general gas constant. *τ*(*T*) is the surface non-uniformity parameter. *τ*_ref_ is the surface non-uniformity parameter at *T*_ref_. *χ* and *α* are parameters that determine the temperature dependence of *q*_∞,p_(*T*), and *τ*(*T*).

The adsorption of CO_2_ by this adsorbent conforms to the phenomenon described by the linear driving force model (LDF) [45], which is used to describe its adsorption kinetics, as shown in Equation (17).

(17) $$\frac{dq}{dt}=k(q_{\mathrm{eq}}-q)$$

where q is the adsorption capacity at the current moment.

## 5. Initial and Boundary Conditions

The initial conditions are assumed as follows:

(18) $$\left\{\begin{array}{c} T_{i}\left|t=0=\right.T_{i,\mathrm{ini}}\\ x_{i}\left|t=0=\right.x_{i,\mathrm{ini}}\\ P\left|t=0=\right.P_{\mathrm{ini}}\\ q\left|t=0=\right.q_{\mathrm{ini}} \end{array}\right.$$

where, *i* is taken as medium such as g, a, met and hf or gas component such as N_2_, O_2_, CO_2_ and H_2_O. The boundary conditions of the adsorption process are represented by Equation (19):

(19) $$\left\{\begin{array}{c} m_{f,g}\left|z=0=\right.m_{f,g}^{\mathrm{in}}\\ m_{f,\mathrm{hf}}\left|y=0=\right.m_{f,\mathrm{hf}}^{\mathrm{in}}\\ \frac{\partial P_{CO_{2}}}{\partial z}\left|z=0=\right.\frac{\partial P_{CO_{2}}}{\partial z}\left|z=\mathrm{out}=\right.0\\ \frac{\partial T_{g}}{\partial z}\left|z=0=\right.\frac{\partial T_{g}}{\partial z}\left|z=\mathrm{out}=\right.0\\ \frac{\partial T_{\mathrm{hf}}}{\partial y}\left|y=0=\right.\frac{\partial T_{\mathrm{hf}}}{\partial y}\left|y=\mathrm{out}=\right.0 \end{array}\right.$$

The boundary conditions of evacuation process are represented by Equation (20):

(20) $$\left\{\begin{array}{c} \frac{\partial m_{f,g}}{\partial z}\left|z=0=\frac{\partial m_{f,g}}{\partial z}\left|z=out=\right.\right.0\\ \frac{\partial P_{CO_{2}}}{\partial z}\left|z=0=\right.0\\ \frac{\partial \sum P_{i}}{\partial z}\left|z=out=\right.\alpha_{p,eva}\left(P_{pump}-\sum P_{i}\right)\\ \frac{\partial T_{g}}{\partial z}\left|z=0=\right.\frac{\partial T_{g}}{\partial z}\left|z=out=\right.0\\ \frac{\partial T_{hf}}{\partial y}\left|y=0=\right.\frac{\partial T_{hf}}{\partial y}\left|y=out=\right.0 \end{array}\right.$$

The boundary conditions of heating process are represented by Equation (21):

(21) $$\left\{\begin{array}{c} \frac{\partial m_{f,g}}{\partial z}\left|z=0=\right.\frac{\partial m_{f,g}}{\partial z}\left|z=out=\right.0\\ \frac{\partial P_{CO_{2}}}{\partial z}\left|z=0=\right.\frac{\partial P_{CO_{2}}}{\partial z}\left|z=out=\right.0\\ \frac{\partial T_{g}}{\partial z}\left|z=0=\right.\frac{\partial T_{g}}{\partial z}\left|z=out=\right.0\\ T_{hf}\left|y=0=T_{des}\right.\\ \frac{\partial T_{hf}}{\partial y}\left|y=out=\right.0 \end{array}\right.$$

The boundary conditions of desorption process are represented by Equation (22):

(22) $$\left\{\begin{array}{c} \frac{\partial m_{f,g}}{\partial z}\left|z=0=\frac{\partial m_{f,g}}{\partial z}\left|z=out=\right.\right.0\\ \frac{\partial P_{CO_{2}}}{\partial z}\left|z=0=\right.0\\ \frac{\partial \sum P_{i}}{\partial z}\left|z=out=\right.\alpha_{p,pro}\left(P_{pump}-\sum P_{i}\right)\\ \frac{\partial T_{g}}{\partial z}\left|z=0=\right.\frac{\partial T_{g}}{\partial z}\left|z=out=\right.0\\ T_{hf}\left|y=0=T_{des}\right.\\ \frac{\partial T_{hf}}{\partial y}\left|y=out=\right.0 \end{array}\right.$$

### Solution Method

**Figure 12:**
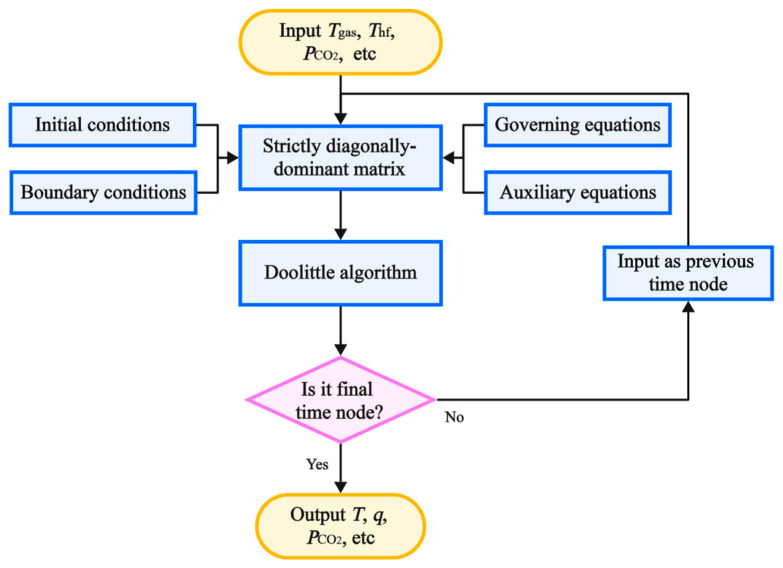
Method for solving the model.

In this study, the simulation results are calculated with the help of the mathematical calculation software MATLAB R2022b. As shown in Figure 12, the backward difference method is employed to obtain a numerical solution of the partial differential equation system. According to previously defined solving conditions, this problem falls under a mixed initial-value and boundary-value problem. The reactor bed is spatially meshed. Divide the grid along the direction of heat transfer, fluid flow direction, and air blowing direction, respectively. At each grid, convert the derivative into a difference quotient, thereby achieving discretization. Combined with the auxiliary equations, the control equation system is adjusted to a strictly diagonally dominant matrix, which is then solved by the Doolittle algorithm. It is worth noting that to avoid the huge computational volume and slow computational speed of the iterative method, the adsorption/desorption rate term adopts the value of the previous time step as an approximation. The time step is taken as 5 s.

## 6. Validation

**Figure 13:**
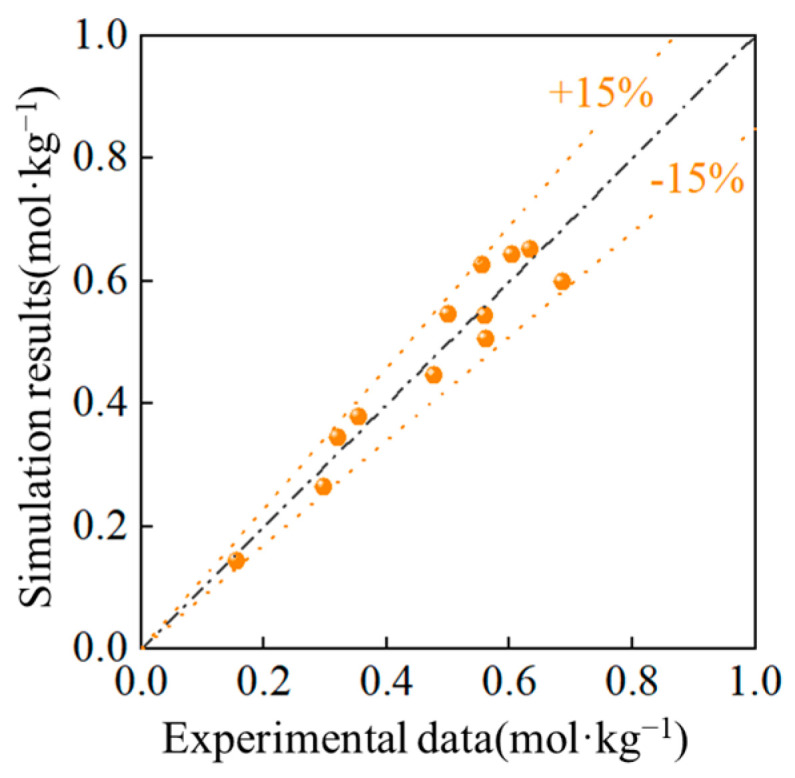
Error range between simulation results and experimental data.

Compare the simulation results with the experimental data. Multiple sets of experiments under different operating conditions have been conducted, and the CO_2_ capacity results are summarized in Figure 13. The error of the model can be controlled within 15%. In this work, the comparison between experiment and simulation by CO_2_ capacity, and it is enough to prove the accuracy because, in the DAC cycle, the capacity of the adsorbent is concerned with ad/desorption temperature and desorption pressure. If the capacities of simulation and experiment are consistent, we can conclude that the temperature and pressure results of simulation are accurate. Thus, in this study, it can be concluded that the model has good accuracy and predictive ability.

## 7. Conclusions

This paper studies the adsorption-based DAC system using mathematical simulation methods; the main conclusions are summarized as follows:

1. A structured DAC reactor incorporating an internal heat source is designed, and a three-dimensional dynamic mathematical model suitable for the TVSA operation is constructed.

2. The temporal and spatial distribution of temperature and adsorption capacity within the reactor are quantified using the proposed model. The temperature responds rapidly over time, and the difference in adsorption capacity remains within 0.12 mol·kg^−1^ spatially.

3. Parametric analyses of the heat source temperature and vacuum pressure during the regeneration process are conducted. Under the optimal operating conditions of 90 °C and 1 kPa, the system achieves an annual CO_2_ productivity of 125 tCO_2_·year^−1^. The corresponding mechanical and thermal energy consumptions are 4.72 GJ·tCO_2_^−1^ and 11.91 GJ·tCO_2_^−1^, respectively.

4. For different adsorbents, the adsorption isotherms are obtained through the characterization of the actual materials. Based on the test results of adsorption isotherms and the fitting of the isotherm model, the corresponding mathematical expressions were obtained, exactly as described in Equation (13). In this way, the DAC performance of different adsorbents in the reactor of this study can be obtained.

5. The reactor structure offers good heat and mass transfer performance, thus ensuring uniform distribution of temperature and adsorption capacity during ad/desorption processes. Besides, with an internal cooling source, higher desorption temperature, and lower desorption pressure, the cycle capacity in TVSA increases, which is a promising method to enhance DAC performance in applications.

## Figures and Tables

**Table 1:** Structure parameters of the reactor.

Parameters	Value
Specification of reactor (m × m × m)	1.7 × 1.7 × 1.7
Specification of an adsorbent sheet (m × m × m)	1.6 × 1.6 × 0.022
Number of adsorbent sheets	40
Number of parallel heat exchange tubes	50
Number of fins	400
Mass of adsorbent (kg)	21

**Table 2:** Performance parameters of adsorbent.

Parameter	Value	Unit
*b*_ref,c_	0.0019	kPa^−1^
*b*_ref,p_	9.4 × 10^−17^	kPa^−1^
*E*_a_	3.09	kJ·mol^−1^
*q*_∞,c_	3.6	mol·kg^−1^
*q*_∞,ref_	2.92	mol·kg^−1^
*T*_ref_	298.15	K
−Δ*H*	105.1	kJ·mol^−1^
*α*	1.96	
*τ*_ref_	0.404	
*χ*	2.48	

## Data Availability

The original contributions presented in this study are included in the article. Further inquiries can be directed to the corresponding authors.
